# Supplementary material for: Diagnostic Potential of Exosomal microRNAs in Colorectal Cancer
Source: Diagnostics (Basel). 2022 Jun 8;12(6):1413. doi: 10.3390/diagnostics12061413 (PMC9221658; doi:10.3390/diagnostics12061413)
Supplement: Supplementary file 1 [file diagnostics-12-01413-s001.zip › diagnostics-1613692-supplementary/diagnostics-1613692-supplementary.pdf]

Table S1: Characteristics of chosen miRNA.

miRNA	Biological Role	Targeted Genes	Correlation of Expression in CRC
**Let7**	tumor suppression	HMGA2, KRAS, c-MYC,	higher expression was associated with higher CRC-specific mortality
**mir16**	tumor suppression	cox-2, zyxin	low expression associated with poor survival of CRC patients
**mir19**	cell survival, proliferation, differentiation and angiogenesis	TG2	high expression associated with poorer survival
**mir21**	cellular proliferation and differentiation	PDCD4, NFIB, SPRY2	associated with poor survival and response to chemotherapy
**mir23**			Increased expression associated with liver metastasis
**mir29**	cell proliferation, cell cycle, cell senescence, differentiation, and apoptosis	CDK6, MCL1, BCR/ABL1	correlated with a poor prognosis in patients with CRC
**mir34**	tumor suppression	p53	increased expression associated with poor cancer-specific mortality
**mir92**	cell proliferation, suppress apoptosis of cancer cells, induce tumor angiogenesis	integrin α5,E-cadherin, Bim	high expression in CRC patients was associated with poor survival
**mir222**		PTEN, p57Kip2, PDLIM2	correlated with liver metastasis, distant metastasis and shorter overall survival rate of patients with CRC
**mir451**	tumor suppression	MIF, IL6R, Ywhaz	downregulation correlated with cancer persistence or recurrence after surgery

Table S2. Characteristics of chosen primers (*all* Qiagen GmbH, Hilden, Germany).

miScript Primer Assay	miR Base	miRNA Sequence
Hs_miR-23a_2	MIMAT0000078	5'AUCACAUUGCCAGGGAUUUCC
Hs_miR-451_1	MIMAT0001631	5'AAACCGUUACCAUUACUGAGUU
Hs_miR-34a*_1	MIMAT0004557	5'CAAUCAGCAAGUAUACUGCCCU
Ce_miR-39_1	MIMAT0000010	5'UCACCGGGUGUAAAUCAGCUUG
Hs_miR-92_1	MIMAT0000092	5'UAUUGCACUUGUCCCGGCCUGU
Hs_miR-16_1	MIMAT0000069	5'UAGCAGCACGUAAAUAUUGGCG
Hs_miR-21_2	MIMAT0000076	5'UAGCUUAUCAGACUGAUGUUGA
Hs_let-7c_1	MIMAT0000064	5'UGAGGUAGUAGGUUGUAUGGUU
Hs_miR-222_2	MIMAT0000279	5'AGCUACAUCUGGCUACUGGGU
Hs_miR-19a_1	MIMAT0000073	5'UGUGCAAAUCUAUGC AAAACUGA
Hs_miR-29a_1	MIMAT0000086	5'UAGCACCAUCUGAAAUCGGUUA
Hs_SNORD43_11	MS00007476	5’CACAGATGATGAACTTATTGAC

Table S3. Median levels of Cel-miR-39 with standard deviations.

Cel-miR-39	Healthy	Adenoma	CRC
**exosomes**	15.23 ± 2.14	13.46 ± 3.17	13.89 ± 1.87
**serum**	28.87 ± 2.87	25.45 ± 2.34	27.51 ± 3.33

Table S4. Details of median-normalized miRNA values in the exosomal compartment, shown in Figure 2.

miRNA	Diagnosis	Patients	Outliers	min.	25% Quant.	Median	75% Quant.	Max	mean	Standard Deviation	Standard Error
**Let7**	healthy	26	0	−7.33	−4.19	−2.75	−1.51	−0.10	−2.87	1.67	0.33
	adenoma	19	1	−3.82	−2.33	−1.87	−0.88	0.59	−1.66	1.26	0.30
	CRC	22	1	−4.50	−2.50	−2.19	−1.34	−0.19	−2.01	1.03	0.22
**mir16**	healthy	26	0	−6.33	−3.81	−2.26	−0.93	0.78	−2.53	1.96	0.38
	adenoma	20	0	−6.41	−3.52	−2.69	−0.20	1.53	−1.90	2.23	0.50
	CRC	23	0	−4.79	−2.02	−1.56	−0.68	2.79	−1.36	1.48	0.31
**mir19**	healthy	26	0	−7.54	−5.63	−4.49	−3.57	−0.74	−4.44	1.85	0:36
	adenoma	20	0	−6.32	−4.70	−3.81	−2.37	0.01	−3.51	1.84	0.41
	CRC	23	0	−6.83	−4.71	−4.26	−2.80	0.62	−3.77	1.56	0.33
**mir21**	healthy	26	0	−8.27	−6.15	−5.02	−3.66	−0.85	−4.81	1.75	0.34
	adenoma	20	0	−8.52	−3.48	−2.75	−2.31	−0.69	−3.17	1.98	0.44
	CRC	23	0	−7.88	−6.25	−4.35	−2.34	0.73	−4.36	2.30	0.48
**mir23**	healthy	26	0	−7.44	−6.13	−4.44	−3.32	0.02	−4.39	2.12	0.42
	adenoma	20	0	−9.52	−3.36	−2.72	−1.36	0.34	−2.79	2.43	0.54
	CRC	23	0	−6.67	−4.29	−3.46	−2.42	1.49	−3.22	1.71	0.36
**mir29**	healthy	26	0	−6.24	−4.72	−3.54	−2.22	0.03	−3.36	1.78	0.35
	adenoma	19	1	−4.96	−2.48	−1.72	−1.08	0.91	−1.83	1.63	0.38
	CRC	23	0	−5.03	−3.51	−2.88	−1.61	1.84	−2.52	1.50	0:31
**mir34**	healthy	26	0	−7.19	−5.86	−5.33	−3.78	−1.22	−4.73	1.67	0.33
	adenoma	20	0	−8.54	−4.69	−3.85	−2.77	0.94	−3.94	2.30	0.51
	CRC	22	1	−7.11	−5.64	−4.84	−3.23	−1.10	−4.60	1.60	0.35
**mir92**	healthy	26	0	−7.42	−5.09	−3.80	−2.47	−0.09	−3.67	1.81	0.35
	adenoma	20	0	−8.02	−3.57	−2.71	−1.14	1.54	−2.60	2.29	0.51
	CRC	23	0	−5.48	−3.91	−3.07	−1.57	2.01	−2.72	1.69	0.35
**mir222**	healthy	26	0	−7.34	−5.95	−5.01	−4.10	−1.09	−4.77	1.66	0.33
	adenoma	20	0	−8.06	−4.04	−3.04	−2.29	−0.38	−3.29	1.93	0.43
	CRC	23	0	−6.87	−5.13	−4.58	−2.85	0.66	−3.99	1.67	0.35
**mir451**	healthy	26	0	−5.79	−3.15	−2.49	−1.06	0.92	−2.23	1.73	0.34
	adenoma	20	0	−7.06	−3.46	−2.79	0.45	1.78	−1.82	2.34	0.52
	CRC	23	0	−4.01	−2.27	−1.90	−0.40	2.98	−1.37	1:55	0.32

Table S5. Areas under the curve (AUC) and sensitivities (SN) at 95% specificity (SP) for the different miRNAs in the exosomal compartment. Comparisons of healthy and non-healthy (adenoma or CRC) patients were made. *p*-value of Let7 = 0.009, of mir16 = 0.061 and of mir23 = 0.012.

miRNA	Healthy vs. Non-Healthy (Adenoma or CRC)	
	**AUC**	**SN@95%SP**
**Let7**	0.67	38.5%
**mir16**	0.62	23.1%
**mir19**	0.61	23.1%
**mir21**	0.65	3.8%
**mir23**	0.69	34.6%
**mir29**	0.68	26.9%
**mir34**	0.59	3.8%
**mir92**	0.65	11.5%
**mir222**	0.68	15.4%
**mir451**	0.59	23.1%

Table S6. Median-normalized miRNA values in serum. Comparisons of different diagnoses (healthy controls, adenoma patients, carcinoma patients) were made. Differences are shown as *p*-values. *p*-values of < 0.05 (highlighted with *) were considered to be statistically significant.

miRNA	Healthy vs. Adenoma	Healthy vs. CRC	Adenoma vs. CRC
**Let7**	<0.001 *	0.296	0.005 *
**mir16**	<0.001 *	0.533	<0.001 *
**mir19**	<0.001 *	0.193	0.056
**mir21**	<0.001 *	0.619	<0.001 *
**mir23**	<0.001 *	0.541	<0.001 *
**mir29**	<0.001 *	0.742	<0.001 *
**mir34**	<0.001 *	0.289	0.013 *
**mir92**	<0.001 *	0.275	0.002 *
**mir222**	0.017 *	0.310	0.241
**mir451**	<0.001 *	0.173	0.010 *

Table S7. Details of median-normalized miRNA values in serum.

miRNA	Diagnosis	Patients	Outliers	min.	25% Quant.	Median	75% Quant.	max	mean	Standard Deviation	Standard Error
**Let7**	healthy	26	0	−7.51	−1.57	−0.27	2.04	4.89	−0.13	2.98	0.58
	adenoma	20	0	0.03	1.67	3.17	5.08	6.22	3.30	1.95	0.44
	CRC	23	0	−4.33	−2.02	0.94	4.07	5.97	0.84	3.40	0.71
**mir16**	healthy	26	0	−2.81	3.82	4.66	5.68	10.26	4.89	2.78	0.55
	adenoma	20	0	5.25	7.37	8.69	9.86	11.30	8.59	1.81	0.40
	CRC	23	0	1.06	2.51	4.61	8.61	10.65	5.46	3.47	0.72
**mir19**	healthy	25	1	−11.10	0.92	2.16	3.34	5.67	1.14	4.20	0.86
	adenoma	19	0	−3.11	3.33	5.88	7.43	11.41	5.41	3.56	0.82
	CRC	23	0	−5.11	0.45	1.72	6.17	11.18	2.87	4.80	1.00
**mir21**	healthy	26	0	−3.37	1.85	3.37	4.73	7.95	3.38	2.55	0.50
	adenoma	20	0	2.68	5.55	8.70	9.54	11.87	7.74	2.55	0.57
	CRC	23	0	−2.29	0.47	3.53	7.00	11.38	3.90	4.31	0.90
**mir23**	healthy	26	0	−3.44	3.38	4.41	5.97	12.60	4.53	3.14	0.62
	adenoma	20	0	4.00	6.35	8.68	10.73	12.80	8.72	2.83	0.63
	CRC	23	0	−6.74	−0.50	2.92	8.47	12.92	3.73	5.48	1.14
**mir29**	healthy	25	1	−0.94	0.29	1.30	1.95	4.79	1.45	1.71	0.35
	adenoma	20	0	1.00	2.68	4.26	5.89	8.25	4.47	2.36	0.53
	CRC	23	0	−2.48	−0.97	0.37	3.47	7.62	1.22	2.91	0.61
**mir34**	healthy	26	0	−10.32	−6.56	−4.73	−2.92	−1.20	−4.73	2.28	0.45
	adenoma	20	0	−3.78	−2.59	−1.45	−0.25	3.96	−0.95	2.35	0.53
	CRC	23	0	−7.75	−6.68	−6.07	−1.68	3.81	−3.66	4.27	0.89
**mir92**	healthy	26	0	−7.54	−1.54	−0.01	1.71	4.77	0.04	2.57	0.50
	adenoma	20	0	0.24	2.04	3.38	4.88	6.26	3.54	1.79	0.40
	CRC	23	0	−2.89	−1.57	−0.63	3.46	6.25	0.95	3.15	0.66
**mir222**	healthy	26	0	−7.57	−1.98	−0.40	0.65	4.76	−0.44	2.44	0.48
	adenoma	20	0	−1.89	−0.23	0.90	2.83	6.25	1.46	2.63	0.59
	CRC	23	0	−3.32	−1.77	−0.69	2.29	6.59	0.40	3.19	0.67
**mir451**	healthy	25	0	−3.54	4.49	5.86	7.76	12.38	6.04	3.55	0.71
	adenoma	16	0	5.98	9..51	12.04	13.35	17.36	11.48	3.39	0.85
	CRC	23	0	1.06	4.39	6.19	10.84	16.65	7.80	5.04	1.05

Table S8. Details for tumor markers CEA and CA 19-9.

Marker	Diagnosis	Patients	Min.	25% Quant.	Median	75% Quant.	Max.	Mean	Standard Deviation	Standard Error
**CEA**	healthy	24	0.00	0.00	0.35	1.10	4.08	0.76	1.07	0.22
	adenoma	17	0.00	0.00	1.10	2.35	4.53	1.40	1.40	0.34
	CRC	22	0.00	0.69	1.10	1.61	3.40	1.24	0.88	0.19
**CA19-9**	healthy	24	0.00	0.69	1.70	2.97	5.36	1.87	1.51	0.31
	adenoma	16	0.00	1.52	2.77	3.30	5.17	2.50	1.42	0.35
	CRC	21	0.00	2.40	2.64	3.09	5.46	2.73	1.16	0.25
